# Metabolic transition of milk triacylglycerol synthesis in response to varying levels of palmitate in porcine mammary epithelial cells

**DOI:** 10.1186/s12263-018-0606-6

**Published:** 2018-07-06

**Authors:** Yantao Lv, Shihai Zhang, Wutai Guan, Fang Chen, Yinzhi Zhang, Jun Chen, Yang Liu

**Affiliations:** 1 Guangdong Provincial Key Laboratory of Animal Nutrition Control, College of Animal Science, South China Agricultural University, Guangzhou, 510642 People’s Republic of China; 2 College of Animal Science and National Engineering Research Center for Breeding Swine Industry, South China Agricultural University, Guangzhou, 510642 People’s Republic of China; 3 Agro-Biological Gene Research Center, Guangdong Academy of Agricultural Sciences, Guangzhou, 510640 People’s Republic of China

**Keywords:** Palmitate, Porcine mammary epithelial cells, Milk fat biosynthesis, Lipogenic genes

## Abstract

**Background:**

Milk in mammals is a key source of lipids for offspring, providing both critical energy and essential fatty acids. For lactating sows, palmitic acid is one of the most abundant fatty acids in milk, providing 10~12% of the suckling pig total dietary energy supply. However, the effects of exogenous palmitic acid on milk fat synthesis in sow mammary glands are not well-known. In this study, we investigated the effects of palmitic acid on lipogenic genes in porcine mammary epithelial cells (pMECs) to explore the role of exogenous palmitic acid in mediating milk triacylglycerols (TAG) synthesis.

**Methods:**

Porcine mammary epithelial cells were cultured for 24 h in the presence of different concentrations of palmitate (0, 25, 50, 100, 200, 400, and 600 μM). The effect of palmitate on cell viability was tested via MTT assay. Intracellular lipid accumulation was measured through Oil Red O staining, and TAG levels were quantified by enzymatic colorimetric methods. Expression of genes and proteins involved in milk fat biosynthesis were assayed with quantitative real-time polymerase chain reaction (*q*PCR) and Western blotting, respectively.

**Results:**

Incubation with palmitate promoted cellular lipid synthesis in a dose-dependent manner, as reflected by the increased TAG content and enhanced formation of cytosolic lipid droplets. The increased lipid synthesis by palmitate was probably attributable to the upregulated mRNA expression of genes associated with milk fat biosynthesis, including long-chain fatty acid uptake (*LPL*, *CD36*), intracellular activation and transport *(ACSL3*, *FABP3*), TAG synthesis (*GPAM*, *AGPAT6*, *DGAT1*), lipid droplet formation (*PLIN2*), and regulation of transcription (*PPARγ*). Western blot analysis of CD36 and DGAT1 proteins confirmed the increased lipid synthesis with increasing incubation of palmitate. However, the genes involved in fatty acid de novo synthesis (*ACACA*, *FASN*), fatty acid desaturation (*SCD*), and regulation of transcription (*SREBP1*, *INSIG1*) were inversely affected by incubation with increasing concentrations of palmitate. Western blot analysis of ACACA protein confirmed this decrease associated with increasing levels of palmitate.

**Conclusions:**

Results from this study suggest that palmitate stimulated the cytosolic TAG accumulation in pMECs, probably by promoting lipogenic genes and proteins that are involved in lipid synthesis. However, addition of palmitate decreased the fatty acid de novo synthesis in pMECs.

## Background

Mammalian milk fat is composed of 98% triacylglycerols (TAG), which are composed of three fatty acids esterified to a three-carbon glycerol backbone [1]. The three most abundant fatty acids in sow milk are palmitic acid (C16:0, 33 wt%, on average), oleic acid (C18:1, 32 wt%, on average), and linoleic acid (C18:2, 14 wt%, on average) [2]. Palmitic acid represents 20~30% of the fatty acids in human and sow milk and ~ 70% of palmitic acid is esterified at the sn-2 position of the milk TAG [3–6]. Palmitic acid in milk contributes 10~12% of the total dietary energy supply of the suckling offspring [1]. The palmitic acid in the mammary gland has two main origins: (1) de novo synthesis by mammary epithelial cell and (2) release into the circulation from chylomicrons by lipoprotein lipase (LPL) or from adipose tissue by hormone-sensitive lipase (HSL) [7]. Because the palmitic acid from the two main origins is approximately equal, palmitic acid uptake by the mammary gland is an efficient means to modify milk fat content and fatty acid composition in lactating animals. For example, addition of 8% palm oil (rich in palmitic acid and oleic acid) to the sow diet during lactation significantly increased milk fat concentration and total fatty acids, mainly by increasing the concentration of palmitic acid and oleic acid in milk [8]. Gene expression analysis suggests that lipogenesis in the mammary gland can be modulated by a high-fat diet, with increased dietary fat consumption resulting in a marked decrease in fatty acid synthesis and fewer short-chain fatty acids in milk [9–11].

Since palmitic acid is the most abundant saturated fatty acid in sow diets, and the major fatty acid synthesized de novo, it is important to identify lipogenic genes that are regulated by palmitic acid. In sows, there are few reports in the literature available on the effect of exogenous palmitic acid on metabolic pathway related to milk TAG synthesis. We have previously established the developmental pattern of key factors that channel fatty acids towards milk TAG synthesis in porcine mammary tissue during lactation [2]. Milk fat is synthesized and secreted by specialized secretory epithelial cells that line the luminal cavity of mammary alveoli. Therefore, by using porcine mammary epithelial cells (pMECs) as an in vitro model, we investigated the effects of exogenous palmitate on the two primary pathways for milk lipid synthesis to explore the role of exogenous palmitic acid in mediating milk TAG synthesis.

## Methods

### Cell culture

pMECs were isolated from the mammary gland of a lactating Large White sow on day 17 of lactation and cloned using the method described in Zheng and He [12]. Briefly, a 1-cm^3^ sample of mammary gland tissue was dissected with sterile scissors into 1-mm^3^ fragments. The mammary tissue fragments were digested by collagenase II (500 U/mL) (Sigma-Aldrich, St. Louis, MO, USA) for 1 h at 37 °C and 5% CO_2_. The isolated cells were cultured in Dulbecco’s modified Eagle’s medium/F12 (DMEM/F12) (Gibco, Grand Island, NY, USA) media supplemented with 10% fetal bovine serum (FBS) (Gibco, Grand Island, NY, USA), 5 μg/mL Insulin-Transferrin Selenium (ITS) (ScienCell, Carlsbad, CA, USA), 10 ng/mL epidermal growth factor (EGF) (Gibco, Grand Island, NY, USA), 10 ng/mL IGF-1, 5 μg/mL hydrocortisone (Sigma-Aldrich, St. Louis, MO, USA), 100 U/mL penicillin, and 100 μg/mL streptomycin (Gibco, Grand Island, NY, USA) at 37 °C in a humidified atmosphere with 5% CO_2_. Culture medium was changed every 24 h. Fluorescence-activated cell sorting (FACS) analysis for cytokeratin expression in the cells revealed that they were composed of 90% mammary epithelial cells. Additionally, the cells had a high mRNA abundance of *β-casein* using RT-PCR. In this study, pMECs from the 11th passages were used.

Palmitate (≥ 98.5% pure isomers) (Sigma-Aldrich, St. Louis, MO, USA) used for treatments was conjugated to fatty acid-free bovine serum albumin (BSA) (Equitech-Bio, Kerrville, TX, USA) at a 4:1 ratio.

### Cell viability assay

The effect of palmitate on cell viability was tested via MTT assay. Briefly, pMECs in suspension were seeded at 5 × 10^3^ cells per well in 96-well microtiter plates, and these cells were grown in a humidified atmosphere of 5% CO_2_ in air at 37 °C. Then, the cells were exposed to varying concentrations of palmitate (0, 25, 50, 100, 200, 400, and 600 μM) for 24 h. Subsequently, culture medium was carefully removed and exchanged for fresh medium. Twenty microliters of MTT solution (5 mg/mL PBS) was then added to each well, and plates were incubated at 37 °C for 4 h. During incubation, the active enzymes of the viable cells transformed the yellow MTT into insoluble purple formazan crystals. The top medium was then removed, and DMSO was added to each well to dissolve the formazan crystals. The absorbance of the solution was measured at a wavelength of 490 nm on a multifunctional plate reader.

### Assessment of triglyceride storage

#### Intracellular lipid droplet staining

Intracellular lipid accumulation was measured through Oil Red O staining. pMECs in suspension were seeded at 5 × 10^4^ cells per well in 24-well microtiter plates, and these cells were grown in a humidified atmosphere of 5% CO_2_ in air at 37 °C. Cells were then exposed to varying concentrations of palmitate (0, 25, 50, 100, 200, 400, and 600 μM) for 24 h. Subsequently, the cells were washed with PBS twice, fixed in 4% paraformaldehyde for 30 min at room temperature, and then rinsed with PBS three times (10 min each time). A 0.5% Oil Red O/isopropyl alcohol solution was added for 1 h to the cells, which were then washed several times with PBS. The stained cytoplasmic lipids were visualized and photographed by an inverted microscope at × 400 magnification. Lipid droplet diameter was measured using Image J software (NIH). In each field captured on camera, the mean diameter of the five largest lipid droplets was calculated and used to estimate the maximum diameter of the intracellular lipid droplet.

#### Quantification of the intracellular TAG content

TAG levels were also quantified by enzymatic colorimetric methods using commercial kits (Applygen, Beijing, China). Briefly, pMECs in suspension were seeded at 5 × 10^5^ cells per well in 6-well plates and cultured until 80~90% confluent. Then, the cells were exposed to varying concentrations of palmitate (0, 25, 50, 100, 200, 400, and 600 μM) for 24 h. After that, culture medium was carefully removed, and the cells were rinsed with PBS three times. Total protein samples were homogenized in RIPA lysis buffer (Beyotime, Nanjing, China). After centrifugation at 12,000×*g* for 5 min at 4 °C, the supernatants were collected and stored at − 80 °C until analysis. TAG contents in supernatant were assayed using commercial kits (Applygen, Beijing, China), and protein concentrations in supernatant were determined using a Pierce BCA protein Assay kit (Thermo Fisher Scientific, Waltham, MA, USA). The TAG contents were normalized for protein in each well and expressed as total TAG per cellular protein. Each experiment was performed in triplicate and repeated a minimum of three times.

### RNA extraction and real-time quantitative PCR

pMECs were seeded at 5 × 10^5^ per well in a 6-well plate and cultured until 80~90% confluent. Then, the cells were incubated with increasing concentrations of palmitate (0, 25, 50, 100, 200, 400, and 600 μM) for 24 h. After that, total RNA was isolated from the cells using TRIzol reagent (Invitrogen, Carlsbad, CA, USA) according to the manufacturer’s instructions. Total RNA was purified from contaminating DNA by DNaseItreatment performed on RNeasy columns following the manufacturer’s instructions (Takara, Tokyo, Japan). The purity of RNA (A260/A280) for all samples was 1.8~2.0 via a spectrophotometer, indicating that samples were pure and clean. The integrity of the RNA was also checked by ethidium bromide-stained agarose gel electrophoresis. First-strand cDNA synthesis was performed by using a PrimeScript RT reagent kit with gDNA eraser (Takara, Tokyo, Japan). cDNA was then diluted 1:5 with DNase/RNase-free water.

The mRNA abundance of the reference gene (*β-actin*) and target genes related to lipogenic pathways were determined by quantitative real-time polymerase chain reaction (*q*PCR). All the primer sequences of *q*PCR are shown in Table 1. *q*PCR was performed using 1 μL diluted cDNA combined with 19 μL of a mixture composed of 10 μL 1 × SYBR Green Real-Time PCR Master Mix (Toyobo, Tokyo, Japan), 0.5 μL of 10 μM forward and reverse primer, respectively, and 8 μL DNase/RNase-free water in a 96-well reaction plate (Axygen, Tewksbury, MA, USA). *q*PCR was performed on an ABI Prism 7500 Sequence Detection System using a SYBR® PCR protocol. The PCR protocol was composed of an initial denaturation at 95 °C for 1 min and 40 cycles of amplification comprising denaturation at 95 °C for 15 s, annealing at primer-specific temperatures (58~61 °C) for 15 s and elongation at 72 °C for 20 s. Melting curves were analyzed after the reactions. The specificity of the reaction was monitored by determining the product melting curve to avoid nonspecific signals. The amplification of a single product of the expected size was confirmed using 1.5% agarose gel electrophoresis.

**Table 1:** Characteristics of primers used for real-time quantitative PCR analysis

Gene	NCBI GenBank	Primers^a^	Primer sequence(5′ → 3′)	Amplicon (bp)^b^
*LPL*	NM_214286.1	F.654	ATTGCAGGAAGTCTGACCA	124
		R.777	CGTCTACAAAATCTGCGTC	
*CD36*	DQ192230.1	F.433	GGACTCATTGCTGGTGCTGT	169
		R.601	GTCTGTAAACTTCCGTGCCTGT	
*ACSL3*	NM_001143698.1	F.470	ACCCTGGATGTGATACGCTA	150
		R.619	AGTTCCCAAGAATAACCTTTT	
*FABP3*	AY569332.1	F.157	CTGGGAGTGGAGTTTGATGAGAC	164
		R.320	CCATGGGTGAGTGTCAGGAT	
*ACACA*	NM_001114269.1	F.3638	ACATCCCCACGCTAAACA	186
		R.3823	AGCCCATCACTTCATCAAAG	
*FASN*	NM_001099930.1	F.1884	GCTTGTCCTGGGAAGAGTGTA	115
		R.1998	AGGAACTCGGACATAGCGG	
*SCD*	NM_213781.1	F.785	TGACCTAAAAGCCGAGAA	164
		R.948	GCACGATGGCGTAACGAAGA	
*GPAM*	XM_001927875.1	F.955	ACTATCTCCTGCTCACTTTCA	146
		R.1100	CGTCTCATCTAGCCTCCGTC	
*AGPAT1*	EU282358.1	F.170	CCTTCTACAACGGCTGGAT	174
		R.343	GCTGTGAGGGAGGGAAGTGG	
*AGPAT6*	FJ439669.1	F.56	CTGGGCATCTCCCTGACTGT	198
		R.253	GATTCCATTGGTGTAGGGCTTG	
*DGAT1*	AY116586.1	F.6569	TGGACTACTCACGCATCAT	176
		R.6744	GTGGAAGAGCCAGTAGAAGAA	
*LPIN1*	NM_001130734.1	F.2084	CACATTTTGCCCACCCTT	164
		R.2247	GTGCCACGCTCGTTGACC	
*LPIN2*	NM_001141987.1	F.2026	CCTATGGAACTGGAACGA	141
		R.2166	TTGATGGAGTGGTAGAGCTTGG	
*PLIN2*	NM_214200.2	F.1183	CTCCTCAGTTCCAGCAAG	113
		R.1295	GGATAAAAGGGACCTACCAG	
*SREBP1*	NM_214157.1	F.984	AGCGGACGGCTCACAATG	121
		R.1104	CGCAAGACGGCGGATTTA	
*INSIG1*	NM_001244521.1	F.509	TGTCGTGGGCTTGCTCTA	123
		R.631	GCACTGGCGTGGTTGATG	
*SCAP*	AY705448.2	F.1089	GCGGTGAGATTTTCCCCTAC	185
		R.1273	GCCAATGAGGATGATGCC	
*PPARα*	DQ437887.1	F.323	CAGCGTGGCACTGAACATC	144
		R.466	CTCCGATCACATTTGTCATAGAC	
*PPARγ*	NM_214379.1	F.1170	AGCCCTTTGGTGACTT	213
		R.1382	AGGACTCTGGGTGGTT	

^a^Primer direction (F-forward; R-reverse) and hybridization position on the sequence

^b^Amplicon size in base pair (bp)

All sample mRNA levels were normalized to the values of the reference gene (*β-actin*), and the results were expressed as fold changes of threshold cycle (Ct) value relative to control using the 2^−ΔΔCt^ method [13].

### Western blot analysis

pMECs were cultured until 80~90% confluent and were then incubated with different concentrations of palmitate (0, 25, 50, 100, 200, 400, and 600 μM) for 24 h. After that, cells were collected and homogenized in RIPA lysis buffer (Beyotime, Nanjing, China) for assay of proteins related to lipogenic pathway. The homogenates were combined with equal volumes of SDS sample buffer, and the proteins were separated by electrophoresis on a 5~12% polyacrylamide gel and transferred to nitrocellulose membranes. The membranes were blocked with 5% skim milk in Tris-buffered saline with Tween, followed by overnight probing with the following primary antibodies: (1) CD36 (N-15) antibody (1:500, Santa Cruz Biotechnology, Santa Cruz, CA, USA), (2) ACACA (T-18) antibody (1:500, Santa Cruz Biotechnology, Santa Cruz, CA, USA), (3) DGAT1 antibody (1:500, Abcam, Cambridge, MA, USA), (4) SREBP1 (C-20) (1:500, Santa Cruz Biotechnology, Santa Cruz, CA, USA), (5) PPARγ (T-18) antibody (1:500, Abcam, Cambridge, MA, USA), and (6) β-actin (C4) antibody (Santa Cruz Biotechnology, Santa Cruz, CA, USA). β-actin was included as a loading (internal) control. After washing, membranes were incubated with secondary antibody (ABR, Golden, CO, USA). The chemiluminescent signal was detected by using ECL reagents (Beyotime, Nanjing, China), and bands were quantified by Image Processing Software (Image Pro Plus 6.0).

### Statistical analysis

Data were analyzed using General Linear Model procedure of SAS software (SAS Version 9.0) as a completely randomized design. Regression analysis was performed to evaluate linear and quadratic effects of palmitate on the various response criteria. Differences at *p* < 0.05 were considered statistically significant. Values are expressed as means ± SEM.

## Results

### Cell viability

**Fig. 1:**
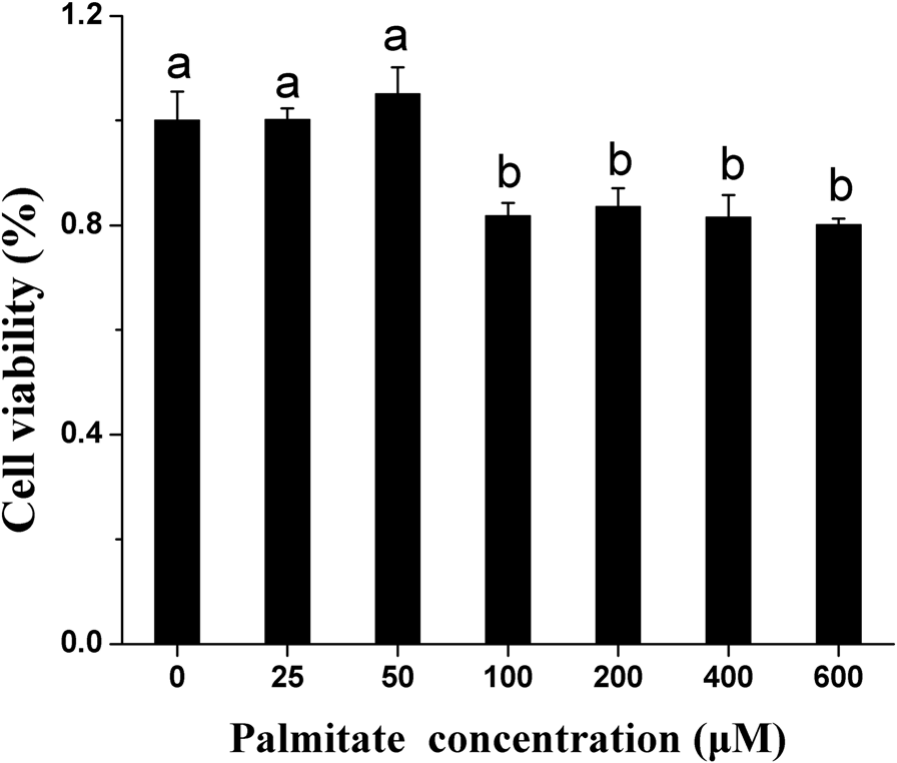
Effect of palmitate on cell viability in pMECs. pMECs were incubated for 24 h with different concentrations of palmitate (0 (control), 25, 50, 100, 200, 400, and 600 μM). Cell viability was estimated by MTT test. Values, expressed as percentage of control, are presented as mean ± SEM (*n* = 6). Different letters indicate statistical significance between different concentrations of palmitate treatment groups (*p* < 0.05)

Incubation with 0~50 μM palmitate for 24 h did not affect the viability of pMECs, but exposure to 100~600 μM palmitate decreased the cell viability by approximately 20% (Fig. 1). This indicates that cell viability tended to be suppressed when the palmitate concentration was above 50 μM.

### Accumulation of intracellular TAG

**Fig. 2:**
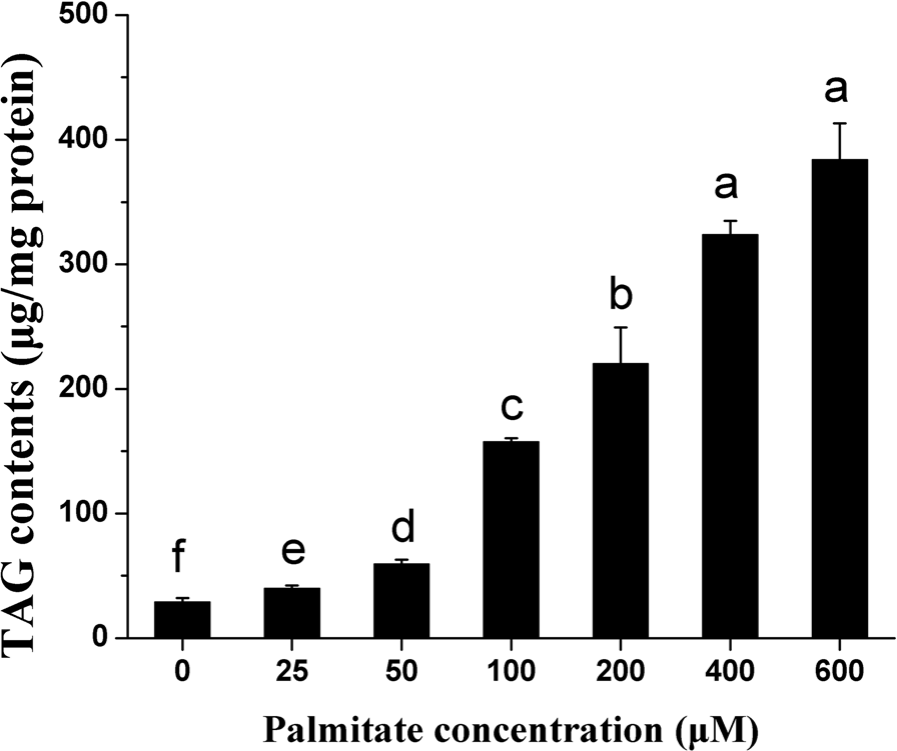
Effect of palmitate on cellular TAG content in pMECs. pMECs were incubated for 24 h with different concentrations of palmitate (0 (control), 25, 50, 100, 200, 400, and 600 μM). The data are expressed as the mean ± SEM (*n* = 6). Different letters indicate statistical significance between different concentrations of palmitate treatment groups (*p* <  0.05)

**Fig. 3:**
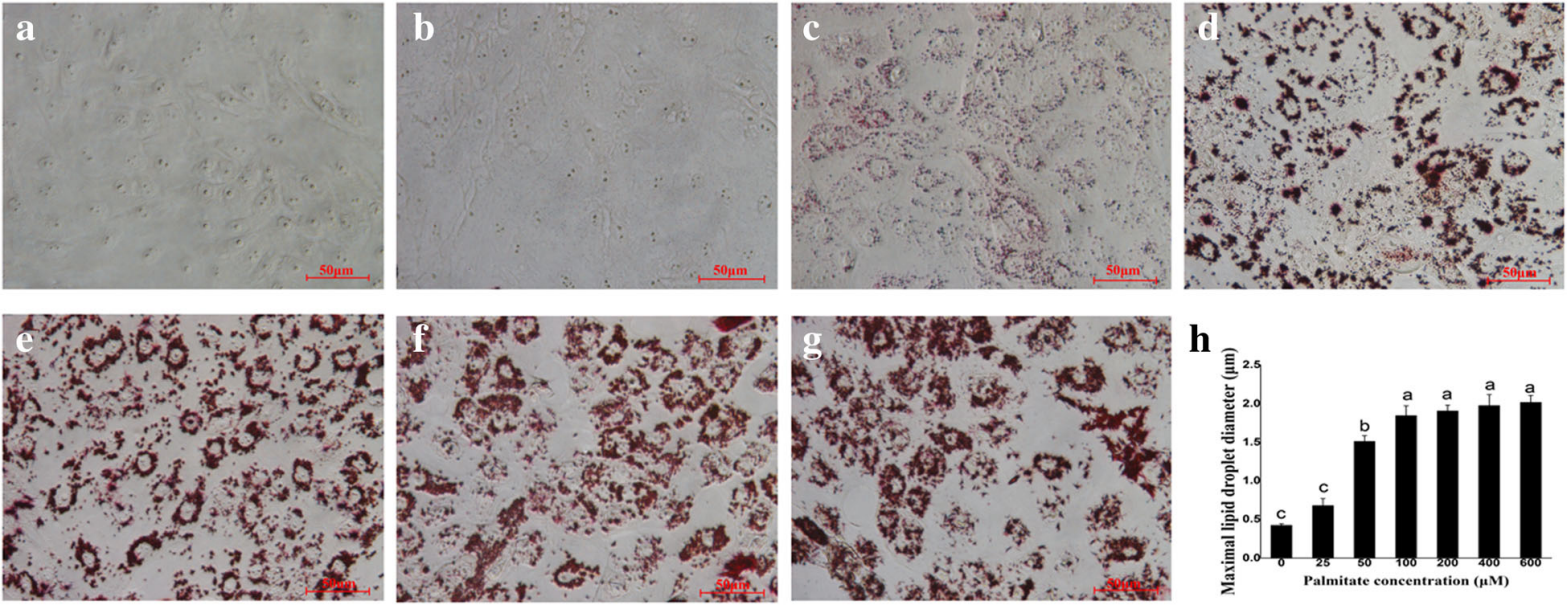
Effect of palmitate on lipid droplet formation in pMECs. Cells were incubated for 24 h with different concentrations of palmitate (0 (control), 25, 50, 100, 200, 400, and 600 μM) and then stained with Oil Red O and visualized by light microscopy with × 400 magnification. **a** 0 (control); **b** 25 μM; **c** 50 μM; **d** 100 μM; **e** 200 μM; **f** 400 μM; **g** 600 μM. **h** Maximal lipid droplet diameter. In **h**, data are expressed as mean ± SEM (*n* = 5) and different letters indicate statistical significance between different concentrations of palmitate treatment groups (*p* < 0.05)

The addition of palmitate in the medium for 24 h significantly increased cellular TAG contents in a dose-dependent manner (Fig. 2). Similarly, Oil Red O staining confirmed the enhanced formation of cytosolic lipid droplets in pMECs when incubated with increasing concentrations of palmitate (Fig. 3a–g). The average diameter of large lipid droplets was increased linearly or quadratically with increasing palmitate (*p* < 0.05), with the maximal value observed at 100~600 μM (Fig. 3h). These results indicate that exogenous palmitate increased cytosolic TAG accumulation and lipid droplets formation in pMECs.

### Upregulating the expression of genes or proteins associated with long-chain fatty acid uptake, intracellular activation, and transport in pMECs

**Fig. 4:**
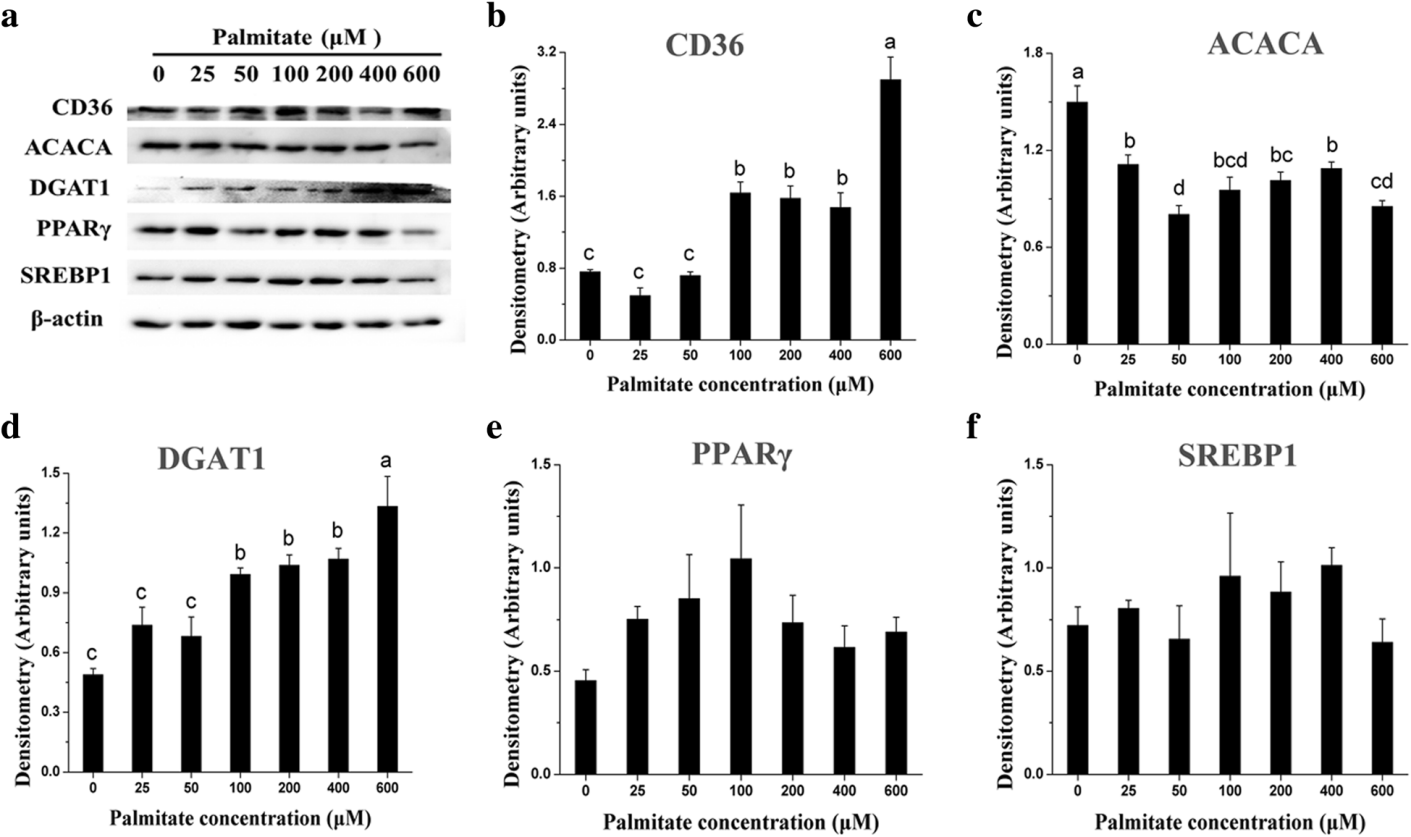
Effect of palmitate on the expression of proteins involved in lipid synthesis in pMECs. pMECs were incubated for 24 h with different concentrations of palmitate (0 (control), 25, 50, 100, 200, 400, and 600 μM). **a** Representative protein bands of CD36, ACACA, DGAT1, PPARγ, and SREBP1. Bar graph of protein expression level relative to β-actin, including CD36 (**b**), ACACA (**c**), DGAT1 (**d**), PPARγ (**e**), and SREBP1 (**f**). The data are expressed as the mean ± SEM (*n* = 3). Different letters indicate statistical significance between different concentrations of palmitate treatment groups (*p* < 0.05)

Incubation with 25~600 μM palmitate for 24 h upregulated the expression of genes associated with long-chain fatty acid (LCFA) uptake (*LPL*), intracellular activation (*ACSL3*), and transport (*CD36*) in pMECs (Table 2). Particularly, cellular *LPL* and *ACSL3* mRNA expression were increased linearly or quadratically with increasing palmitate (*p* < 0.05), with the maximal values observed at 200~400 μM (Table 2). *CD36* and *FABP3* mRNA expression in pMECs was increased linearly with increasing palmitate from 50 to 600 μM (*p* < 0.05), with the highest values at 600 μM palmitate (Table 2). Consistent with its gene mRNA expression, cellular CD36 protein expression was significantly upregulated by 100~600 μM palmitate (*p* < 0.05), with the highest values at 600 μM palmitate (Fig. 4b).

**Table 2:** Effect of palmitate on mRNA expression of genes involved in lipid synthesis in pMECs

Gene	Palmitate concentration (μM)							SEM	*P* value		
	0	25	50	100	200	400	600		Palmitate	Linear	Quadratic
Fatty acid uptake and import into cells											
*CD36*	1.00^d^	2.11^cd^	2.63^bc^	1.91^cd^	2.48^bc^	3.49^b^	4.73^a^	0.14	< 0.001	< 0.0001	0.10
*LPL*	1.00^c^	1.96^bc^	2.47^abc^	2.32^abc^	3.74^a^	2.67^ab^	2.17^bc^	0.17	0.03	0.02	0.01
Fatty acid activation and intra-cellular transport											
*ACSL3*	1.00^e^	1.44^d^	1.75^cd^	1.98^bc^	1.97^bc^	2.58^a^	2.19^b^	0.05	< 0.0001	0.0001	0.02
*FABP3*	1.00^d^	1.17^d^	1.73^c^	2.36^b^	2.05^bc^	2.35^b^	3.50^a^	0.06	< 0.0001	< 0.0001	0.26
Fatty acid de novo synthesis and desaturation											
*ACACA*	1.00^a^	1.15^a^	1.01^a^	0.48^b^	0.52^b^	0.65^b^	0.52^b^	0.04	< 0.001	< 0.0001	0.22
*FASN*	1.00^a^	0.96^a^	0.53^b^	0.52^b^	0.43^b^	0.46^b^	0.34^b^	0.03	< 0.0001	< 0.0001	< 0.01
*SCD*	1.00^ab^	0.64^abc^	0.34^cd^	0.27^d^	0.55^bcd^	0.55^bcd^	1.13^a^	0.06	0.01	0.62	< 0.001
TAG synthesis and lipid droplet formation											
*AGPAT1*	1.00	1.21	1.16	1.03	1.16	1.51	1.41	0.05	0.11	0.02	0.42
*AGPAT6*	1.00^c^	1.50^b^	1.34^bc^	1.37^bc^	1.39^bc^	2.25^a^	2.13^a^	0.06	< 0.01	< 0.0001	0.21
*LPIN1*	1.00^ab^	0.85^bc^	0.69^bc^	0.80^bc^	0.48^c^	1.31^a^	0.75^bc^	0.05	0.03	0.94	0.14
*LPIN2*	1.00^b^	1.08^b^	0.84^bc^	0.94^bc^	0.70^c^	1.93^a^	2.10^a^	0.04	< 0.0001	< 0.0001	< 0.0001
*DGAT1*	1.00^d^	1.80^c^	1.80^c^	1.61^c^	2.56^b^	2.17^bc^	3.28^a^	0.07	< 0.0001	< 0.0001	0.27
*GPAM*	1.00^b^	1.25^b^	1.49^b^	1.57^b^	1.57^b^	2.81^a^	2.42^a^	0.10	< 0.01	< 0.0001	0.50
*PLIN2*	1.00^c^	0.90^c^	1.48^c^	1.55^c^	2.70^b^	3.37^b^	5.52^a^	0.18	< 0.0001	< 0.0001	< 0.01
Regulation of transcription											
*SREBP1*	1.00^ab^	1.03^a^	0.61^c^	0.68^c^	0.66^c^	0.76^bc^	0.53^c^	0.03	< 0.01	< 0.001	0.16
*PPARa*	1.00	1.12	0.90	1.11	0.92	0.87	1.02	0.05	0.78	0.57	0.89
*PPARγ*	1.00^c^	2.03^bc^	1.80^bc^	0.91^c^	1.73^bc^	2.83^ab^	4.73^a^	0.27	0.03	< 0.01	0.05
*INSIG1*	1.00^abc^	1.06^abc^	0.84^bcd^	0.73^cd^	0.52^d^	1.21^ab^	1.38^a^	0.05	< 0.01	0.10	< 0.001
*SCAP*	1.00^c^	1.43^a^	1.21^b^	1.03^c^	0.98^c^	1.40^a^	1.20^b^	0.02	< 0.0001	0.29	0.49

Different letters indicate statistical significance between different concentrations of palmitate treatment groups (*p* < 0.05)

### Downregulating the expression of genes or proteins related to fatty acid de novo synthesis and desaturation in pMECs

Incubation with palmitate for 24 h suppressed the expression of genes associated with fatty acid de novo synthesis and desaturation in pMECs. mRNA expression of *ACACA*, *FASN*, and *SCD* in pMECs were decreased linearly or quadratically with increasing palmitate (*p* < 0.05; Table 2). The mRNA expression of *ACACA* in pMECs was downregulated by 100~600 μM palmitate but not affected by 25~50 μM palmitate. Cellular mRNA abundance of *FASN* with 50~600 μM palmitate was 45~65% lower than control. *SCD* mRNA expression at 100 μM was 71% lower than the control (Table 2). The protein expression of ACACA in pMECs was significantly downregulated by palmitate (*p* <  0.05) (Fig. 4c).

### Influencing the expression of genes or proteins related to TAG synthesis and lipid droplet formation in pMECs

Incubation of pMECs with palmitate for 24 h increased the mRNA expression of genes associated with TAG synthesis (*GPAM*, *AGPAT6*, *DGAT1*) and lipid droplet formation (*PLIN2*) (Table 2). The cellular mRNA expression of *GPAM*, *AGPAT6*, *DGAT1*, and *PLIN2* were increased linearly or quadratically with increasing palmitate (*p* < 0.05; Table 2). Similarly, the DGAT1 protein expression in pMECs was significantly upregulated by 100~600 μM palmitate (*p* < 0.05), with the highest values observed at 600 μM palmitate (Fig. 4d).

### Influencing the expression of genes or proteins related to regulation of transcription in pMECs

*PPARγ* mRNA expression in pMECs was increased linearly or quadratically with increasing palmitate (*p* < 0.05), with maximal values observed at 600 μM. In contrast to *PPARγ*, the mRNA expression of *SREBP1* and *INSIG1* were decreased linearly or quadratically with increasing palmitate (*p* < 0.05; Table 2), with minimum values observed at 600 or 200 μM palmitate, respectively. *SCAP* mRNA expression in pMECs was increased by 25~50 μM or 400~600 μM palmitate but decreased by 100~200 μM palmitate (*p* < 0.05). The cellular *PPARα* mRNA expression was not affected by palmitate. The protein expressions of PPARγ and SREBP1 in pMECs were not affected by palmitate (Fig. 4e, f).

## Discussion

In this study, we found that ≤ 50 μM palmitate did not affect the viability of pMECs (Fig. 1), but higher concentrations (≥ 100 μM) of palmitate decreased the cell viability. It was evident that the optimal concentration of palmitate for pMECs viability ranged from 0~50 μM. Similar to our results, it was reported in bovine mammary epithelial cells (bMECs) that cell proliferation tended to be suppressed when palmitate was above 100 μM [14]. Although the reason for suppression of cell viability with high concentrations of palmitate has not been elucidated in this study, the mechanism by which palmitate causes cell death has been reported in other types of cells to be related to oxidative injury [15–17].

In the current study, we also found that the addition of exogenous palmitate to pMECs increased cytosolic TAG contents in a concentration-dependent manner and enhanced the formation of lipid droplets. It was notable that the high concentration of palmitate enhanced lipid synthesis even when the cell viability was suppressed. Consistent with our results, it was reported that addition of 200~600 μM palmitate to bMECs enhanced the accumulation of cellular TAG [16, 18–20]. TAG and other neutral lipids are stored in cytoplasmic lipid droplets, the immediate precursors of milk lipids [21]. We observed that the larger droplets in the palmitate treatment were associated with higher cellular TAG content in pMECs.

We also evaluated the promotive effect of palmitate on TAG synthesis and potential mediation through regulation of lipid synthesis-related pathways, including fatty acid de novo synthesis and uptake of exogenous fatty acid. It is known that mammary cells take up LCFA from albumin-bound fatty acids and lipoproteins, and this process is dependent on membrane hydrolase and fatty acid transport proteins. The enzyme LPL is located in the capillary lumen in the mammary gland and functions to hydrolyze circulation-derived TAG into free fatty acids. Subsequently, the free fatty acids are transported into cells by transport proteins. CD36 is the main protein responsible for exogenous LCFA trans-membrane transport in lactating mammary glands [2, 22, 23]. Our previous study demonstrated that both *LPL* and *CD36* have higher mRNA expression as early as the onset of milk synthesis in the lactating porcine mammary gland [2]. In this study, we observed that exogenous palmitate enhanced the expression of *LPL* mRNA in pMECs (Table 2), although it has been reported that *LPL* mRNA expression in bMECs is not affected by addition of palmitate [24]. Similarly, palmitate enhanced cellular CD36 expression at both the mRNA and protein level (Fig. 4b). Consistent with our results, it was reported that palmitate increased the cellular *CD36* mRNA expression in bMECs [18, 20]. Our results, and reports in the literature, indicate that provision of exogenous palmitate to mammary epithelial cells can activate intracellular LCFA uptake.

LCFA is activated by ACSL to bind an acyl coenzyme A (CoA) before it is used to synthesize TAG. FABP facilitates the cytosolic transport of both long-chain saturated and unsaturated fatty acids. We previously found that *ACSL3* and *FABP3* were the major isoforms within each gene family in lactating porcine mammary tissue, and their mRNA abundance was upregulated during lactation [2]. In the current study, the mRNA expressions of *ACSL3* and *FABP3* in pMECs were increased linearly or quadratically with increasing concentration of palmitate added (25~600 μM). These results are in accordance with previous reports showing that exposure of bMECs to palmitate enhanced the cellular expression of *FABP3* [24]. However, another independent in vitro study showed that *FABP3* mRNA expression in bMECs was inhibited by addition of palmitate in the culture medium [20]. Elucidation of the reasons for inconsistent regulation of FABP3 in bMECs and pMECs by palmitate will require additional research. Our results suggest that palmitate exerts a positive effect on LCFA uptake (*LPL* and *CD36*), fatty acid activation (*ACSL3*), and intracellular transport (*FABP3*).

FASN and ACACA are considered the crucial enzymes of cellular fatty acid de novo synthesis in the mammary gland, which have been reported to be the primary source of short- and medium-chain fatty acids (almost all C4:0~C14:0 and approximately 50% of palmitic acid) of milk [25, 26]. In this study, the fatty acid de novo synthesis in pMECs was suppressed by 50~600 μM palmitate, as reflected by the downregulated genes for *ACACA* and *FASN*. This is in agreement with previous work in bMECs showing that palmitate suppressed fatty acid de novo synthesis [20]. Suppressive hepatic de novo lipogenesis was also observed in high-fat diet-fed mice [27]. Similarly, it was reported that, in in vivo studies, dietary supplementation of palmitic acid to dairy cows decreased the concentrations of milk de novo synthesized fatty acids (C6:0~C14:0) probably through suppressing the expression of *ACACA* and *FASN* [28–30]. Our results indicated that the fatty acid de novo synthesis in pMECs was suppressed by a higher concentration of exogenous palmitate. The suppressed expression of genes related to fatty acid de novo synthesis was probably due to the palmitate-induced downregulated upstream regulator (SREBP1). However, the reduced cell viability by palmitate at a higher concentration may contribute, to some extent, to the suppression of fatty acid de novo synthesis especially when cells are exposed to ≥ 100 μM palmitate. We also found that palmitate addition suppressed desaturation of LCFA in pMECs, as reflected by the downregulated mRNA expression of *SCD*, the key enzyme responsible for inducing the double bond at Δ^9^ location of saturated fatty acids [31].

The ultimate synthesis of TAG involves the transfer of acyl-fatty acid to the activated glycerol backbone, the process of which is facilitated by acyltransferases, including glycerol-3-phosphate acyltransferase (GPAM), 1-acylglycerol-3-phosphate O-acyltransferases (AGPAT), and diacylglycerol O-acyltransferase (DGAT1) [32]. Additionally, LPIN1 encodes a phosphohydrolase enzyme that catalyzes the dephosphorylation of phosphatidic acid to yield diacylglycerol. PLIN2 (adipophilin) is located on the droplet surface and is associated with lipid droplet storage and control of cellular lipolytic activity [33, 34]. The transcripts of *AGPAT1*, *LPIN1/2*, *DGAT1*, and *PLIN2* are the most abundant transcripts within each specific gene family in the lactating porcine mammary gland [2]. In this study, the mRNA expressions of genes associated with TAG synthesis (*GPAM*, *AGPAT1/6*, *LPIN2*, *DGAT1*) and lipid droplet formation (*PLIN2*) in pMECs were upregulated or tended to be upregulated by palmitate, which is consistent with the promotive effect of palmitate on cellular TAG synthesis and lipid droplets formation. Similar results have been reported by Kadegowda et al. [24] in bMECs.

It has been shown in previous studies that sterol regulatory element binding protein-1 (SREBP1) and PPARγ are of importance in transcriptional regulation of many genes related to milk fat synthesis and secretion and therefore control fatty acid synthesis and uptake in mammary cells [24, 35–38]. In this study, palmitate increased *PPARγ* mRNA expression in pMECs. This indicates that palmitate regulates TAG synthesis probably through activating *PPARγ* and target lipogenic genes, since most LCFAs, including palmitate, are natural ligands and bind to PPARγ and therefore can modulate gene expression and rates of lipogenesis [39, 40]. A recent in vitro study in a liver cell line showed that palmitate modulated the expression of miR-122 and miR-370, which are involved in lipogenesis [41]. This report revealed a new molecular mechanism mediating palmitate-induced TAG synthesis. However, we found that palmitate decreased the cellular mRNA expression of *SREBP1* in pMECs, which is in accordance with a report showing that 100 μM LCFA downregulated the expression of *SREBP1* in bMECs [24]. Our results indicate that palmitate suppresses fatty acid de novo synthesis-related genes (*ACACA* and *FASN*) probably via SREBP1 regulation. SREBP1 has been reported as a key regulator for upregulating genes that encode proteins (ACACA and FASN) involved in fatty acid de novo synthesis in mammary epithelial cells [38].

It can be assumed that the mammary epithelial cells prefer to synthesize milk lipids through uptake of exogenous LCFA rather than through de novo fatty acid synthesis provided that cells have access to abundant palmitate. This assumption was supported by a recent study showing that proliferating fibroblasts prefer to take up palmitate from the extracellular environment rather than synthesizing it de novo [42]. A previous in vivo study showed that feeding high-fat diets promoted hepatic lipid accumulation in mice, and this effect was mainly due to an increased hepatic elongation of palmitate rather than to elongation of de novo synthesized palmitate [43]. Another independent study also demonstrated that, in high-fat diet-fed mice, most hepatic TAG was formed from the re-esterification of existing or ingested lipids but not de novo lipogenesis [27]. Based on our results, we concluded that when exogenous palmitate is provided in the culture media at physiological concentrations, the uptake of extracellular LCFA plays a major role in enhanced TAG synthesis and lipid formation in pMECs, while fatty acid de novo synthesis accounts for a minor fraction of intracellular TAG.

### Implication

Milk fat is an important component of sow milk and provides a large proportion of both calories and essential fatty acids (EFAs) required for the newborn [44]. The lactating porcine mammary gland is estimated to produce approximately 8 kg of milk containing 5% fat per day [3]. Since TAG accounts for > 90% of milk fat, the mammary gland synthesizes about 400 g of TAG daily or nearly 8.4 kg fat during 21 days of lactation. Because modern sows have lower feed intake during lactation, the dietary energy source should be formulated to support this high level of milk fat production [45], prevent sow’s tissue mobilization, and maximize long-term productivity. Given that mammary epithelial cells prefer exogenous LCFA to synthesize TAG, the dietary addition of optimal amounts of fat to support lipid synthesis from two origins may represent the most efficient way for promoting milk fat synthesis. In practical production, addition of 3~5% fat to the sow’s lactation diet can actually increase fat and energy output in sow milk and improve growth performance of nursing piglets [46–48]. Of course, the form of fatty acids (e.g., saturated or unsaturated, number of carbon) should also be considered in practical production.

## Conclusions

**Fig. 5:**
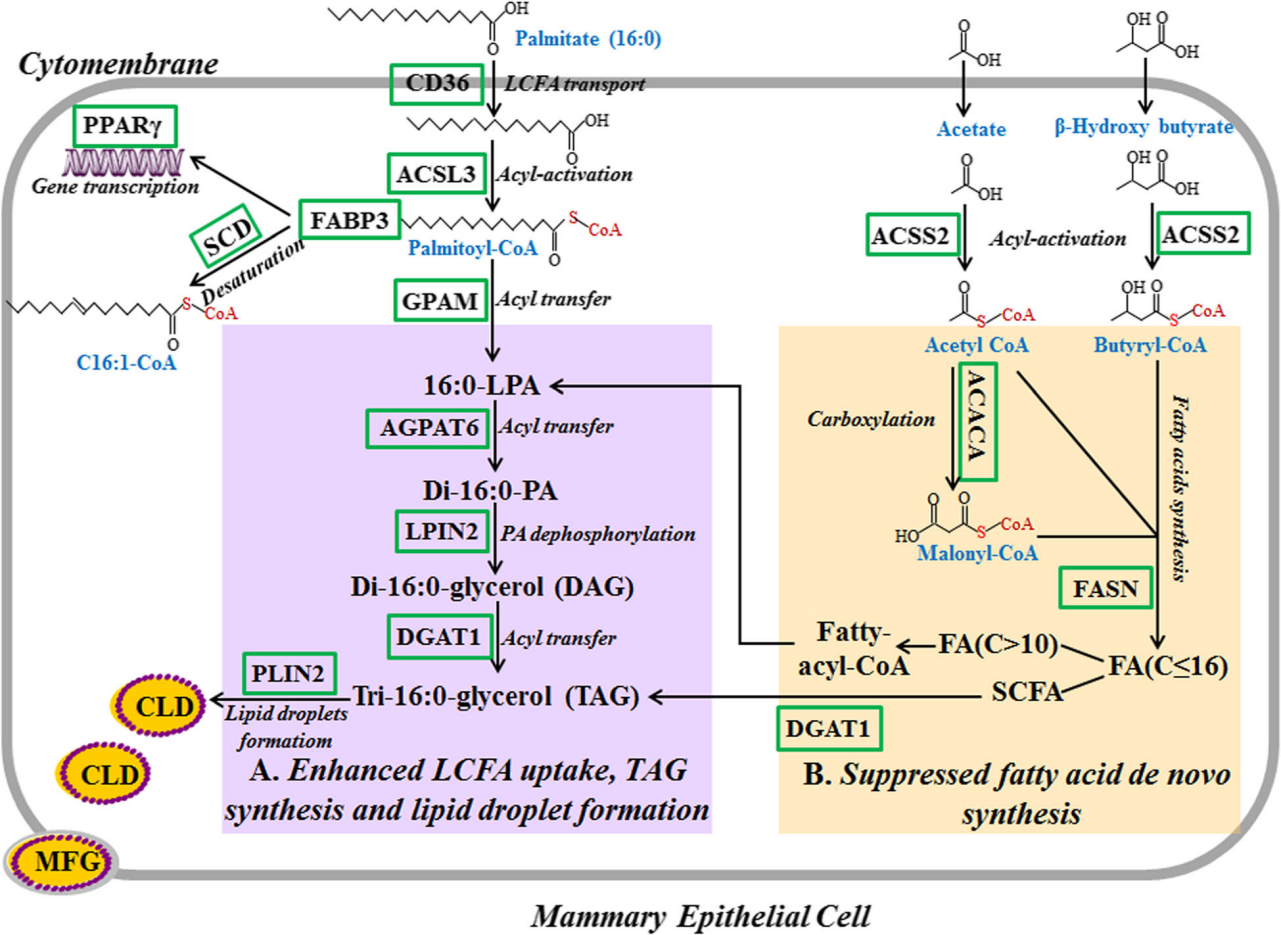
Scheme summarizing interrelationships among cellular pathways regulating lipid synthesis by palmitate in pMECs. **a** Palmitate enhanced the uptake of exogenous LCFA, TAG synthesis, and lipid droplet formation. Uptake of LCFA in pMECs, exampled by palmitate (16:0), was enhanced by palmitate through activating transport proteins (mainly CD36). Cytosolic 16:0 is converted into its activated form (16:0-CoA), with the help of ACSL. Cytosolic 16:0-CoA is transported to endoplasmic reticulum membrane by FABP and esterified there to glycerol-3-phosphate to produce 16:0-LPA by GPAM. In endoplasmic reticulum, addition of a second 16:0-CoA produces di-16:0-PA; di-16:0-PA can be hydrolyzed with LPIN to form a di-16:0-glycerol (DAG). The sn-3 position of DAG is then acylated to form TAG by DGAT. Newly formed TAG forms cytoplasmic lipid droplet in the ER membrane via incorporation. Then, the cytoplasmic lipid droplet was transported to the apical membrane and eventually released. **b** Palmitate suppressed the fatty acid de novo synthesis. In mammary cell, short- and medium-chain fatty acids (almost all C4:0~C14:0 and approximately 50% of palmitic acid) were highly dependent on the de novo synthesis. A series of cytosolic enzymes are required to facilitate this process, of which FASN and ACACA are considered the crucial enzymes of cellular fatty acid de novo synthesis in the porcine mammary gland. ACACA carboxylates acetyl-CoA to form malonyl-CoA, which is further converted by FASN to fatty acids (C ≤ 16). Then, the synthesized fatty acids participate in the TAG formation in endoplasmic reticulum

In summary, our results indicate that palmitate enhanced the cytosolic TAG accumulation in a dose-dependent manner. This is probably because palmitate can regulate the channeling of fatty acids towards milk TAG synthesis and secretion in pMECs though activating the PPARγ pathway and upregulating the target genes associated with milk fat biosynthesis, including LPL and CD36 (LCFA uptake); ACSL3 and FABP3 (intracellular activation and transport); GPAM, AGPAT6, and DGAT1 (TAG synthesis); and PLIN2 (lipid droplet formation) (Fig. 5a). Furthermore, palmitate treatment inhibits milk fatty acid de novo synthesis through suppressing ACACA and FASN gene (fatty acid de novo synthesis) expression (Fig. 5b).

## Abbreviations

ACACA: Acetyl-CoA carboxylase 1
ACSL3: Acyl-CoA synthetase long-chain family member 3
ACSS2: Acyl-CoA synthetase short-chain family member 2
AGPAT1/6: 1-Acylglycerol-3-phosphate O-acyltransferase 1/6
bMECs: Bovine mammary epithelial cells
CD36: Fatty acid translocase/CD36
CLD: Cytoplasmic lipid droplet
DAG: Diacylglycerol
DGAT1: Diacylglycerol acyltransferase 1
FABP3: Fatty acid binding protein 3
FASN: Fatty acid synthase
GPAM: Glycerol-3-phosphate acyltransferase, mitochondrial
HSL: Hormone-sensitive lipase
INSIG1: Insulin-induced gene 1
LCFA: Long-chain fatty acid
LPA: Lysophosphatidic acid
LPIN1/2: Lipin 1/2
LPL: Lipoprotein lipase
MFG: Milk fat globule
PA: Phosphatidic acid
PLIN2: Perilipin 2
pMECs: Porcine mammary epithelial cells
PPARα/γ: Peroxisome proliferator-activated receptor alpha/gamma
PUFA: Polyunsaturated fatty acids
SCAP: Sterol response element binding protein cleavage-activating protein
SCD: Stearoyl-CoA desaturase
SCFA: Short-chain fatty acid
SREBP1: Sterol regulatory element binding protein 1
TAG: Triacylglycerol
